# Mental Health, Well-Being, and Adolescent Extremism: A Machine Learning Study on Risk and Protective Factors

**DOI:** 10.1007/s10802-023-01105-5

**Published:** 2023-08-03

**Authors:** E. F. Haghish, Milan Obaidi, Thea Strømme, Tore Bjørgo, Cato Grønnerød

**Affiliations:** 1 Department of Psychology, University of Oslo, Oslo, Norway; 2 Department of Psychology, Copenhagen University, Copenhagen, Denmark; 3 Centre for the Study of Professions, Oslo Metropolitan University, Oslo, Norway

**Keywords:** Extremism prevention, Violent radicalism, Adolescents, Supervised machine learning, Risk and protective factors

## Abstract

We examined the relationship between adolescents’ extremist attitudes with a multitude of mental health, well-being, psycho-social, environmental, and lifestyle variables, using state-of-the-art machine learning procedure and nationally representative survey dataset of Norwegian adolescents (N = 11,397). Three key research questions were addressed: 1) can adolescents with extremist attitudes be distinguished from those without, using psycho-socio-environmental survey items, 2) what are the most important predictors of adolescents’ extremist attitudes, and 3) whether the identified predictors correspond to specific latent factorial structures? Of the total sample, 17.6% showed elevated levels of extremist attitudes. The prevalence was significantly higher among boys and younger adolescents than girls and older adolescents, respectively. The machine learning model reached an AUC of 76.7%, with an equal sensitivity and specificity of 70.5% in the test dataset, demonstrating a satisfactory performance for the model. Items reflecting on positive parenting, quality of relationships with parents and peers, externalizing behavior, and well-being emerged as significant predictors of extremism. Exploratory factor analysis partially supported the suggested latent clusters. Out of the 550 psycho-socio-environmental variables analyzed, behavioral problems, individual and social well-being, along with basic needs such as a secure family environment and interpersonal relationships with parents and peers emerged as significant factors contributing to susceptibility to extremism among adolescents.

## Introduction

Although extremism does not have a universal definition (Davies, 2008), it can be defined, arguably, as supporting or engaging in violence to bring about a fundamental sociopolitical change that conflicts with the existing order (Khosrokhavar, 2017). However, there is a large step from supporting or justifying the use of violence for political purposes and the actual involvement in violent acts (Schuurman, 2020). This study centers on extremist attitudes, not engagement in extremist violence. Recent studies highlight a rising trend of extremism among adolescents, and as a result, understanding what motivates some adolescents to extremist activities is a pressing societal matter (Harpviken, 2020). On the one hand, adolescents actively explore group relationships and undergo the process of developing identity, sense of significance in life, and socio-political ideologies (Bhui et al., 2012; Harpviken, 2020; Koehler, 2021a). Moreover, they have a high online footprint (Anderson & Jiang, 2018) and are frequently exposed to online extremist contents (Costello et al., 2020; Hawdon et al., 2019). On the other hand, adolescents show limited skills in recognizing extremist content (Assimakopoulos et al., 2017; Nienierza et al., 2021) and are actively targeted online by extremist groups (Bloom, 2019; Ekman, 2014; Nienierza et al., 2021), rendering it imperative to identify risk factors that contribute to adolescents’ susceptibility to extremism as well as those that provide protection against them.

Scholars have proposed a variety of factors influencing the onset of extremism, which can be categorized into three theoretical domains (Obaidi et al., 2023c). The first domain is based on psycho-social models of collective action, such as the need for belonging, identity formation, relative deprivation, and the impact of peers and social networks (Obaidi et al., 2018a, b, c; Tausch et al., 2011). The subsequent domain is grounded in socio-economic and political factors contributing to extremist beliefs, underpinned by sociological theories of collective action (e. g., Pape, 2003). Lastly, the third domain emphasizes individual-level factors such as personality traits (Obaidi et al., 2023a, b, c). However, none of these factors have been adequate in isolation to explain the emergence of extremism (Sageman, 2014). Further, the scholarly discourse on extremism has been preoccupied with identifying risk factors, while understating the importance of well-being and protective factors in preventing extremism (Lösel & Bender, 2017). Prior research posits that resilience and well-being are crucial preventive buffers against extremism among adolescents (Benjamin et al., 2021; Koirikivi et al., 2021). Hence, investigating adolescent extremist attitudes calls for a holistic approach (Bjørgo, 2016a, b). This is also aligned with the General Strain Theory, where violent extremism is conceptualized as a cumulative risk arising from an array of factors that increase the likelihood of individuals’ engagement in violent extremism (Agnew, 2010), providing a foundation for examining extremism through a machine learning approach. This theory postulates that different types of psycho-socio-environmental strains (e.g., perceived injustice, deprivation, or marginalization) can induce negative emotional responses, and if individuals lack coping mechanisms or social support, they may resort to deviant behaviors or extremist behavior to cope with the strain.

Machine learning algorithms accommodate a multitude of items simultaneously without imposing assumptions about item grouping or their distributions, which is beyond the capacity of traditional statistical models and hypothesis-driven quantitative studies (Ivaskevics & Haller, 2022; Yarkoni & Westfall, 2017). In the current paper we aim to advance previous findings by deploying cutting-edge machine learning methodologies and leveraging a nationally representative dataset, encompassing an extensive array of individual, interpersonal, societal, and environmental risk and protective factors that undergo complex interactions amplifying adolescents’ extremism (Van San et al., 2013). Therefore, we emphasize not only risk factors but also the role of well-being and protective factors against extremism. In the subsequent sections we elaborate on these factors, broadly categorized as individual, psycho-socio-environmental, and well-being factors, although these categories are deeply interconnected.

### Individual-Level Drivers of Extremism

Searching for individual-level drivers of extremism, early psychological research paid excessive attention to mental disorders, personality traits, and traumatic experiences. However, the hypothesized link between mental disorders and extremism was later discarded due to paucity of empirical evidence (Vergani et al., 2020). Nevertheless, recent advancements in the field indicate that we may have been too quick to discard the role of mental disorders in extremism. Indeed, an emerging body of evidence reveals a higher incidence of certain mental disorders among 'lone-wolf' actors compared to those acting in groups (Corner & Gill, 2015; Gruenewald et al., 2013a, b). Yet, our understanding of the relationship between mental health and extremism is often restricted to mental disorders and primarily sourced from studies focusing on violent extremist adult men (Barker & Riley, 2022; Bergen et al., 2015). The limited empirical research on the topics raises uncertainties regarding the generalizability of these findings, particularly when examining adolescents' attitudes as opposed to the actions of extremist adults (Bloom, 2019; Harpviken, 2020; Koehler, 2021b). Recognizing the inadequacy of previous studies that focused on mental disorders to explain extremism, recent studies emphasize the importance of adopting a broader perspective on mental health. This entails considering mental health issues beyond the traditional classifications of mental disorder, including but not limited to internalizing and externalizing problems, substance use, victimization, loneliness, and various dimensions of well-being (Harpviken, 2020; Koehler, 2021b).

### Psycho-Socio-Developmental Risk Factors

It is well-established that child maltreatment and neglect, characterized by the failure to meet a child’s physical, emotional, educational, nutritional, and other basic needs, have lasting effects on development of externalizing problems, delinquency, substance use, mental disorders, and obesity (Baldwin et al., 2016). Similarly, research on adolescent gang involvement indicates that the higher the number of risk factors within immediate environments such as family, peers, and school, the higher the probability of such involvement (Hautala et al., 2016). Deficient socialization is also linked to development of violent delinquency and extremism among adolescents (Agnew, 2010; Koehler, 2021b; Van Der Geest et al., 2009). Corroborating these findings, qualitative research reveals that individuals engaged in extremism report numerous instances of maltreatment, exposure to violence, and other risk factors during childhood. They also frequently report externalizing problems during adolescence, which often precedes their involvement in extremist groups (Bubolz & Simi, 2019; Pedersen et al., 2018; Simi et al., 2016). Studies focusing on white supremacist groups have specifically highlighted the prevalence of conduct and substance use problems among their members, further suggesting a potential link between adolescents’ externalizing problems and future involvement in extremist activities (Bubolz & Simi, 2015; Nivette et al., 2017; Simi et al., 2016).

Although there is no single explanation for how childhood risk factors relate to subsequent extremism (Corner & Gill, 2018; Harpviken, 2020), it is believed that these risk factors do not operate in isolation, but rather interact in a complex manner (Maschi et al., 2008; Simi et al., 2016), resulting in a cumulative risk of violence and extremism (Agnew, 2010; Deater–Deckard et al., 1998). For example, social capital, perceived grievance, early life adversities, and deficient socialization can impact adolescents' identities and ideologies. In six studies across 20 different Western countries, Obaidi et al. (2019) demonstrated that perceived relative deprivation could explain the higher rate of key determinants of extremism among second and third generation Muslim westerners. Beyond identity and ideology, psycho-social variables can also influence adolescents’ lifestyles, in turn facilitating their exposure to extremist content and networks (Boehnke et al., 1998; Harpviken, 2020; Jasko et al., 2017; Lösel et al., 2020). Evidence supports this claim, indicating that adolescents who experience discrimination, violence, and marginalization often exhibit shared interests with extremists and report higher rates of encounters with online extremist content (Costello et al., 2016; Hawdon et al., 2019). Having similar lifestyles and shared interests not only increases the likelihood of exposure to extremist content (Jasko et al., 2017), but also fosters stronger emotional connection between adolescents and extremist groups. For example, online video gamers may interact with extremists on shared platforms and due to shared experiences, mutual interests, and teamwork, they might be at an elevated risk of identifying with extremist ideologies (Koehler et al., 2022).

### Well-Being and Resilience

For some individuals, if not all, extremist ideologies and behaviors can be described as quest for meaning in life (Koehler, 2021a), a theme that resonates with theories of well-being (Ryff & Keyes, 1995). Ross et al. (2020) proposed five components for adolescents’ well-being: 1) good health and optimal nutrition, 2) connectedness, 3) safe and supportive environment, 4) learning, competency, education, skills, and employability, and 5) agency, autonomy, and resilience. There is a negative association between adolescents' well-being and risk-taking behaviors and delinquency, which is attributed to factors such as autonomy, self-regulation, and particularly resilience. Resilience is widely recognized as a crucial factor in preventing extremism as it enables adolescents to steer away from risky behaviors (Avedissian & Alayan, 2021). It plays a significant role in helping individuals resist the allure of extremism and make positive choices, serving as a preventive measure against extremism (Benjamin et al., 2021; Koirikivi et al., 2021). A recent study found that a reduction in violent extremist attitudes during late adolescence could be in part explained by self-control and conflict coping skills, both of which are facets of psychological resilience (Nivette et al., 2022). Therefore, strengthening resilience can reduce support for violent extremism among adolescents (Feddes et al., 2015). In Finland, educational institutions have prioritized the enhancement of adolescents’ resilience and well-being as principal objectives to deter extremism (Benjamin et al., 2021; Koirikivi et al., 2021). Similar initiatives are being enacted in other Nordic countries (Nustad et al., 2020).

There is also evidence that school success—reflecting good learning, competency, and education—is inversely associated with adolescents’ extremism (Boehnke et al., 1998; Lösel et al., 2018). Conversely, poor academic performance can be a critical event leading to identity diffusion for adolescents, which in turn can be linked to extremism (Isenhardt et al., 2021). The literature highlights several protective factors against adolescents’ extremism including resilience, adherence to law, acceptance of authority legitimacy, positive parenting behavior, academic achievement, basic attachment to the society, and the presence of significant non-violent individuals in their lives (Lösel et al., 2018, 2020). Yet, our understanding of how other components of adolescent well-being relate to extremism remains limited, particularly because both well-being and extremism are emerging topics in adolescent studies. It is therefore critical to further investigate the role of well-being and other protective factors to develop more comprehensive models of adolescent extremism and strategies to counteract it.

### Current Study

In the current study, we apply machine learning techniques to a nationally representative survey dataset consisting of 11,397 Norwegian adolescents. We aim to address three research questions: First, can machine learning accurately classify adolescents with extremist attitudes when analyzing a multitude of variables together? Previous research has shown that the challenges associated with identifying adolescents who may be at risk of developing extremism tendencies, even for professionals such as trained youth social workers (Van de Weert & Eijkman, 2019) and thus, the application of machine learning could potentially be an aiding tool in this regard. Second, and more importantly, if the accuracy of the classification model exceeds that of chance, what are the most important predictors of extremism according to the model? Third, can these predictors be grouped together into latent factors that represent either risk or protective factors? Because we intend to carry out item-analysis, grouping the most important predictors into factors reduces the dimensionality of the identified predictors, enhancing our understanding of how they relate to one another. Analyzing 550 variables encompassing different aspects of mental health, well-being, family and school environment, and lifestyle, to the best of the authors’ knowledge, positions this study as the most extensive study on adolescents’ extremism.

## Method

### Ethical Approval

This study has been approved by the ethical committee at the Department of Psychology, University of Oslo (reference #21,534,077).

### Sample

A sample of 11,397 high schoolers (5423 boys, 5645 girls, and 329 undisclosed), ranged in age from 13 to 18 (mean = 15.28, SD = 1.60), signed the informed consent form and participated from four cities in Norway and responded to the battery of questionnaires. Consents were also acquired from their parents. The data collection was carried out from 2016 to 2019, as a part of Ungdata,^1^ a broader data collection scheme that regularly surveys adolescents in Norway. The questionnaires were filled out anonymously and electronically in classrooms. Given the large scale of the data collected from tens of high schools across four different municipalities, we consider this dataset to be representative of Norwegian adolescent population. Of the total sample, 315 (2.7%) students did not respond to violent extremism items and were subsequently excluded from the analysis. Moreover, 1,369 participants (12.0%) who responded with “I don’t know” to the extremism items, instead of selecting from the available 5-point Likert options, were also excluded from the analysis. This decision was based on the possibility of some participants choosing this option in an attempt to provide socially desirable responses (Krumpal, 2013), and our concern that recoding “I don’t know” responses to signify no support for violent extremism might bias the results (Løvlien, 2021; Pedersen et al., 2018). Hence, the analysis dataset included 9,713 participants.

### Measures

All items used in the data analysis as well as their response options are available in the Open Science Framework (OSF) repository of the paper via https://osf.io/42m9z/.

#### Extremism

Adolescents' extremism was assessed using a three-item instrument designed to measure their endorsement or justification for the use of violence for political purposes or to bring about political change (Pedersen et al., 2018). The measure aligns with common measures of support for violent extremism used in prior research and has been employed in youth studies (Obaidi et al., 2018a, b, c; Pedersen et al., 2018; Tausch et al., 2011). Pedersen et al. (2018) reported a high Cronbach’s Alpha of 0.91 for the instrument. The items assessed, “*to what extent do you believe the use of violence can be justified* …” and assessed three scenarios, which were “*to raise awareness about a political issue that many people regard as important*,” “*to achieve political change in Norway today*,” and “*to achieve political change in other places in Europe today*.” In addition to “I don’t know” option, respondents could also express to what extent they agree or disagree with the statement using a 5-point Likert scale ranging from “not at all” (coded as 0) to “very much.” Accordingly, each item was coded on a scale from zero to four, with higher scores indicating greater support for extremism. Due to the high skewness in the distribution of the outcome, Pedersen et al. (2018) dichotomized the response options based on cutoff point of 2.67 for the average score across the three items, which indicates that the respondent nearly “agrees” with violent extremism on all items. We adhere to the same cutoff for dichotomizing the scores.

#### Socio-Demographic, Aspiration, Deprivation, and Living Environment

Participants’ age, gender as well as their socio-economic status was surveyed. The latter was measured using the second edition of Family Affluence Scale (Currie et al., 1997), level of education of father and mother, quality of life, quality of the living environment and participants’ subjective evaluation of their family wealth. Perceived deprivation was operationalized through participants' subjective evaluation of their living environment, their perception of local opportunities for a prosperous life, their expressed desire to relocate, and their appraisal of the suitability of their neighborhood as a conducive environment for child upbringing. Future aspirations were assessed through using a series of Likert scale items that measured optimism towards future academic and career achievements, future unemployment prospects, and expected quality of life.

#### Mental and Somatic Health

A variety of mental health instruments were administered, including Hopkins Symptom Checklist for anxiety and depressive symptoms (Derogatis, 1992), the Depressive Mood Inventory (Kandel & Davies, 1982), the UCLA Loneliness Scale (Franzoi & Davis, 1985), the Adolescents’ Self-Perception Profile (Wichstrøm, 1995), and the Antisocial Behavior Scale (Olweus, 1989). Several items were included to assess externalizing problems, which can be grouped into two main domains. The first domain relates to antagonistic behavior, such as engaging in physical or verbal violence, fighting, bullying, and threats, both online and offline. The other domain is related to impulsiveness, characterized by behaviors such as drug and alcohol use, disregard for one’s health, and engaging in reckless behavior (Kotov et al., 2017). Lifetime traumatic and victimization experiences, including instances of being threatened, and being a victim of verbal, physical, or sexual assault in different context such as in the family, in school, online, or offline were also assessed (Stefansen et al., 2009). Finally, the questionnaire also included items related to somatic health such a variety of physical pain, sleep disturbance, and hospitalization.

#### Media, Online Activities, and Video Gaming

Adolescents' engagement with social media, online activities, video gaming, and a variety of leisure activities such as participation in youth clubs or sports organizations were assessed. For instance, to assess the extent of video gaming, one question asked, "*Have your parents or anyone else ever expressed concern about your gaming habits?*" Participants were also asked to consider whether they concealed certain online or offline activities from their parents. An example question was, "*I try to hide most of the activities I do in my free time from my parents,*" with Likert scale responses ranging from "Very true" to "Not true at all." Additionally, participants also reported whether they have online friends that they have never met in person and whether they tend to chat with strangers on the internet.

#### Physical Activities, Diet, Substance Use

Participants were asked to provide insight into their regular sports and physical activities, memberships in sports clubs, dietary habits, and consumption of a variety of legal and illegal substances. For example, they were asked about the frequency of consuming vegetables and fruits, fast food, energy drinks, or diet soft drinks. One of the items, for instance, assessed “*How many times in the last 7 days have you gone to a burger bar, fast food restaurant, *etc*.?*” and responses could range from “*Never*” to “*6 times or more*.” Similarly, adolescents’ consumption of alcohol, cigarettes, other tobacco products, and illegal substances were assessed. In addition, participants were asked how they obtain their substances and what their attitudes towards substances are.

#### Interpersonal Relationships and Social Well-Being

Several survey items assessed adolescents’ social activities, including their involvement in youth clubs, romantic relationships, relationships with parents and friends, as well as their online friendships, providing a detailed understanding of the social networks of the participants. They were also asked to subjectively evaluate the quality of their relationships with family members, friends, and other individuals in their lives. Additionally, the survey assessed adolescents' attitudes towards their school, perceived quality of education, learning preferences, and their parents' academic expectations. Participants also provided insights on perceived stressors related to their school environment.

### Analysis

#### Procedure

Using stratified random split, 80% of the dataset was used for training the algorithm and the remaining 20% was reserved for testing the best-performing models on an unseen dataset. The Gradient Boosting Machine (GBM) algorithm was trained for developing a classification model that distinguishes between adolescents with elevated levels of extremism from those who express no support for political violence. After evaluating and selecting the best performing model on the test dataset (see below), we used the adjROC R package (Haghish, 2022a) to ascertain the threshold in the Receiver Operating Characteristic (ROC) curve where the model's sensitivity and specificity are equal. Furthermore, we calculated the 95% bootstrap confidence interval for the sensitivity and specificity of the model, using 5,000 bootstrap samples. 

#### Class Imbalance and Model Selection

Machine learning classification models typically function optimally when outcome classes are equally represented. However, in our dataset, participants expressing no extremist views (the negative class) significantly outnumbered those exhibiting an elevated level of extremism (the positive class). This discrepancy led to a class imbalance problem, which can skew both the training and performance evaluation of the model in favor of the negative class (Carrington et al., 2020). To mitigate the class imbalance problem, we implemented a down-sampling technique to artificially reduce the number of instances in the negative class and equalize it with the positive class in the *training dataset only* (Provost, 2000). It is crucial to note that we left the testing dataset untouched. Class imbalance can also complicate the evaluation of models’ performance, rendering classification accuracy an unreliable measure for model evaluation and selection (Chicco, 2017). Instead, we utilized the Area Under Precision-Recall Curve (AUPRC) as a criterion for model evaluation and optimization. AUPRC has been found to be more robust against class imbalance compared to commonly used metrics such as Area Under Curve (AUC) of Receiver Operating Characteristic (ROC) or overall accuracy (Davis & Goadrich, 2006). Subsequently, through a tenfold cross-validation procedure, we ranked the models produced via the fine-tuning procedure. The model with the highest AUPRC was then selected for evaluation on the test dataset.

#### Items’ Importance

There are several methods available to assess the importance of items within a machine learning model. In the case of the Gradient Boosting Machine (GBM) algorithm, an important item refers to a variable (or feature) that, when removed, increases the prediction error. In this study, this was estimated based on the gain in AUPRC during the construction of each decision tree (Gregorutti et al., 2017). As GBM is an ensemble algorithm (i.e., it constructs multiple decision trees), the item importance is the average importance across all constructed decision trees (Greenwell et al., 2018). In order to facilitate the interpretation of item importance, it is common to scale the values between 0 and 1, where higher values indicate higher importance to the model.

An alternative approach to assess the importance of predictors is by utilizing a model agnostic procedure such as SHapley Additive exPlanations (SHAP, Lundberg & Lee, 2017). This method can provide local explanations for item importance, thereby offering greater transparency about how each item is utilized by the model to differentiate adolescents with extremist attitudes. To make SHAP contributions comparable, variables are normalized before computing SHAP contributions. The term "important items" or "important predictors" is used in reference to variables that are influential in the model's predictions. It is important to clarify that this does not necessarily imply any ability to predict the emergence of extremism or establish causal relationships with extremist attitudes, as our analysis is based on retrospective survey data. Moreover, the predictors’ importance is not analogous to correlation or regression coefficients, and thus interpreting them as such should be avoided (for review, see Grömping, 2009).

#### Exploratory Factor Analysis

To identify the most important items related to extremism, we used Exploratory Factor Analysis (EFA) to examine the relationships among these items and to discern whether they reflected common risk or protective factors. As stated in the introduction, the primary objective of EFA in this paper is dimension reduction, which aids the interpretation of important items. The GBM algorithm identifies items that offer unique information contribution to the model. However, these items tend to be diverse, potentially uncorrelated or weakly correlated. If the identified important items can be organized into several clusters that represent common risk factors, it would provide valuable insights into the risk factors associated with extremism for future research. This is particularly of importance because such risk factors would stand out among hundreds of items assessing the psycho-socio-environmental aspects of adolescents' lives.

Prior to performing the EFA, we screened the correlation matrix of the important predictors and performed the Kaiser-Meyer Olkin (KMO) and Bartlett’s test of sphericity to verify the feasibility of EFA analysis. If EFA was deemed feasible, we carry out two separate EFA models: 1) using parallel analysis (Horn, 1965), and 2) using provisional number of factors suggested by the authors, following a subjective grouping of the items. When expectations exist regarding the grouping of the items, it is recommended to conduct separate EFA analyses and compare the results to detect differences in the identified factors (Hair et al., 2010). For both models, we used Maximum Likelihood estimator with varimax rotation. Recently, it has been suggested that for ordinal variables or under lack of strong linear relationships, model performance metrics can be employed for evaluating the EFA model and deciding on number of factors to retain (Finch, 2020). We compared the models’ fitness using the Tucker-Lewis Index (TLI) of factor reliability and the Root Mean Square Error of Approximation (RMSEA). In this regard, better EFA model fitness was defined as having a lower RMSEA— where values near 0.05 indicated a close fit and values below 0.08 were acceptable — and a higher value of TLI, with values above 0.9 indicating a good fit (Bentler & Bonett, 1980; Hu & Bentler, 1999; Xia & Yang, 2019).

#### Missing Data Imputation

The mlim R package, which uses machine learning imputation algorithms, was used for imputing the dataset. The mlim imputation algorithm employs state-of-the-art machine learning to fine-tune a model for imputing each variable (Haghish, 2022b). 

## Results

### Descriptive Analysis

Table 1 presents the demographic characteristics of the sample as well as the prevalence of extremist attitudes based on gender, age (grouped by school year), and socio-economic status. Of the total sample, 17.6% of the adolescents showed elevated level of extremist attitude, supporting use of violence for raising political awareness or achieving a political change either in Norway or elsewhere in Europe. Among this group, 59.6% were boys and 40.4% were girls and the observed gender difference was statistically significant (χ^2^(1) = 95.96, *p* < 0.0001), suggesting that boys in this sample were more likely to hold extremist attitudes. The prevalence of extremist attitudes was higher among junior high school students (20.0%) compared to senior high school students (15.1%). This difference was also statistically significant (χ^2^(1) = 39.03, *p* < 0.0001), indicating that younger adolescents were more likely to exhibit extremist attitudes in this sample. As shown in Table 1, the lower the social class, the higher the prevalence of extremism. Although the difference between low and middle classes was statistically significant (χ^2^(1) = 7.31, *p* = 0.0069), the difference between middle and upper class was not significant (χ^2^(1) = 0.488, *p* = 0.485).

**Table 1:** Demographic characteristics of the study population

Items	Some support for political violence (n = 1,687)	No support for political violence (n = 7,879)
Gender		
Boy	1,005 (21.6%)	3,656 (78.4%)
Girl	682 (13.9%)	4,223 (86.1%)
Age (grouped by school year)		
Junior high school (13–15 years old)	990 (20.0%)	3,961 (80.0%)
Senior high school (16–18 years old)	697 (15.1%)	3,918 (84.9%)
Socio-economic status		
Lower class	632 (19.9%)	2,540 (80.1%)
Middle class	376 (17.0%)	1,840 (83.0%)
Upper class	679 (16.3%)	3499 (83.7%)

### Machine Learning Analysis

Fine-tuning the GBM algorithm, the model that achieved the highest AUPRC was selected for further analysis using the testing dataset. The chosen model performed well, with an AUPRC of 40.6%, AUC of 76.7%, and equal sensitivity and specificity of 70.6%. This means the model was able to correctly identify 70.6% of the adolescents with extremist attitudes and similarly, 70.6% of those who did not display any extremist attitudes. The 95% bootstrap confidence interval for the sensitivity and specificity intersection ranged from 68.0% to 73.0%. In comparison, a random guess based on the prevalence of extremism would yield a sensitivity and specificity intersection of just 50.0%. Therefore, the model's performance far exceeds that of random or chance level of prediction, providing substantial value in predicting and understanding adolescent extremism.

Next, the top 20 important items of extremism among Norwegian adolescents were extracted from the model, which are presented in Table 2, alongside the top 20 items with the highest SHAP contribution. Table 2 shows that the two different procedures for evaluating items’ importance suggest a relatively similar set of items, which is noteworthy, adding more credibility to these findings.

**Table 2:** Top 20 predictors of adolescents’ extremist attitudes according to models’ gain in the loss function and SHAP absolute contributions and their relative importance

**Items**	**Model’s contributions**	**SHAP** **contributions**
**My parents are disappointed with me**	1.00	1.00
**I appreciate my parents’ opinions about music**	0.66	0.93
**I hide my free-time activities from my parents**	0.43	0.62
**How often do you tease or threaten other young people?**	0.41	0.59
**How often have you been in a fight the past 12 months?**	0.35	0.62
**I feel in tune with the people around me**	0.27	0.53
**How much does religion influence your life?**	0.23	0.43
**How often do you eat food bought in kiosk near school?**	0.21	0.47
My parents are worried about my gaming *	0.18	0.38
**How often do you eat whole meal bread?**	0.16	0.34
I have been threatened online the past month	0.15	
**How do you normally get from school?** (Public transport, bike, motorbike, etc.) *	0.15	0.20
It’s OK to drive a vehicle after drinking alcohol	0.15	
How often do you tease or threaten other young people online?	0.14	
**My parents are good role models for using social media**	0.14	0.39
Going walking or hiking in the countryside	0.13	
**How often do you receive alcohol from your parents?**	0.13	0.31
How often do you go hunting?	0.12	
**How many friends would visit you if you were hospitalized?**	0.12	0.36
**My parents want me to study hard**	0.11	0.29
Age		0.34
I think using Snus (oral tobacco) is harmful		0.30
Age at the time of first drinking one unit of alcohol?		0.26
I hide my social media activities from my parents		0.26
My parents know my friends’ parents		0.25

Model’s contributions are items contributing to the model’s gain in loss function, i.e., gain in classification accuracy. SHAP contributions are scaled mean absolute SHAP values, computed for all participants in the testing dataset. Items ranked among the top 20 by both procedures are shown in bold font

*Binary or categorical items

**Fig. 1:**
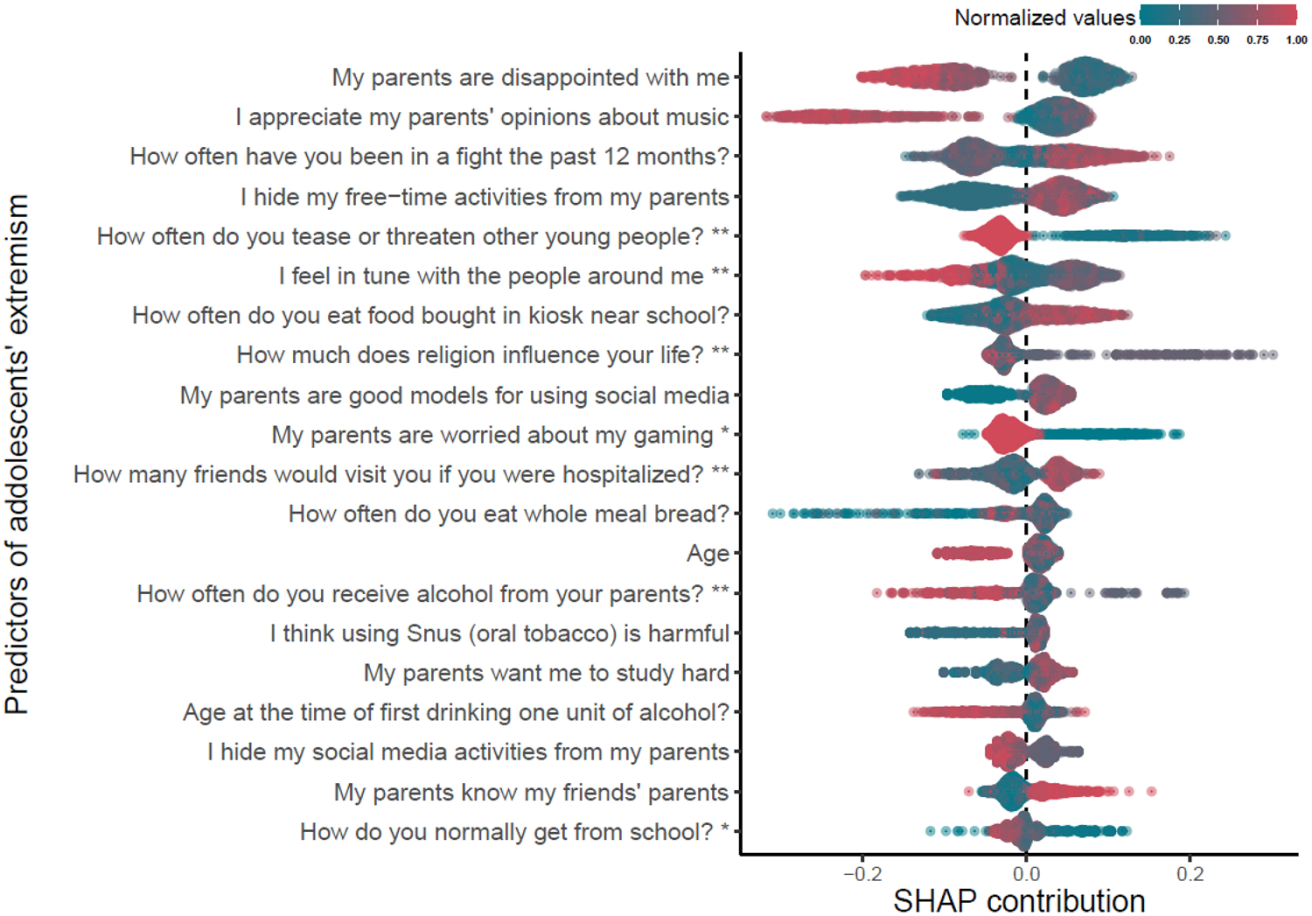
The most important items for classifying adolescents’ extremism based on SHAP contributions. *Binary or categorical items; **items with reversed scoring

Table 2 does not provide any indication that how each item assisted the model’s classification and as noted earlier, these items cannot be interpreted in linear fashion. Figure 1, in contrast, provides insight into how each of the top 20 SHAP contributing items assists the model in distinguishing adolescents with extremist attitudes. This figure is constructed in a way that each bar represents an individual item's impact on the model's classification. The items are ranked in descending order from top to bottom based on their absolute SHAP contributions. The color of each item indicates the normalized values of the item (green for 0, red for 1). To simplify interpretation, all ordinal items are recoded so that lower values (green color) signify full agreement with the statement, and higher values (red color) denote full disagreement with the statement. For items assessing the frequency of a behavior, higher values (red color) indicate more frequent behavior. Therefore, to interpret Fig. 1, the reader should consider both the color of each item as well as the positive or negative value of the SHAP contribution.

For instance, for the item "*my parents are disappointed with me*," which was the most important predictor according to both importance evaluation procedures, positive SHAP values have green color indicating full agreement with the statement. Therefore, agreement with this item provided information for positive classification. In the case of the item “*My parents are worried about my gaming,*” (yes = 0, no = 1), adolescents who agreed (green color) with the statement fed the model with information that helped classifying extremist attitudes and vice versa. Another example is the item “H*ow many friends would visit you if you were hospitalized?*” Here, adolescents who indicated that they had no close friends (red color, signifying no friends) contributed to the model's ability to classify them as having extremist attitudes.

However, some items are more complex and harder to interpret. For example, the item assessing "*How much does religion influence your life?*" which was coded in reverse, does not seem to be unidirectionally related to extremist attitudes. Another example of a complex item is "*How often do you eat whole meal bread?*" While this item significantly contributed to the negative classification with large negative SHAP values, its contribution to the positive classification is less clear. The item "*How often do you eat food bought in a kiosk near school?*" revealed that adolescents who frequently purchased food from a kiosk near their school helped the model classify them as having extremist attitudes. Conversely, those who rarely bought food at a kiosk contributed to the model's ability to identify them as non-extremists. Such types of items, like eating whole meal bread or buying food from a kiosk, should be viewed as proxies for unmeasured latent factors. They provide unique information to the model that is not captured by other items and can reveal certain aspects of adolescents' lives that might be otherwise overlooked. Hence, these lifestyle factors enhance the performance of the classification model, even when numerous psychological, socio-demographic, and environmental factors and risk events are taken into account.

### Exploratory Factor Analyses

**Fig. 2:**
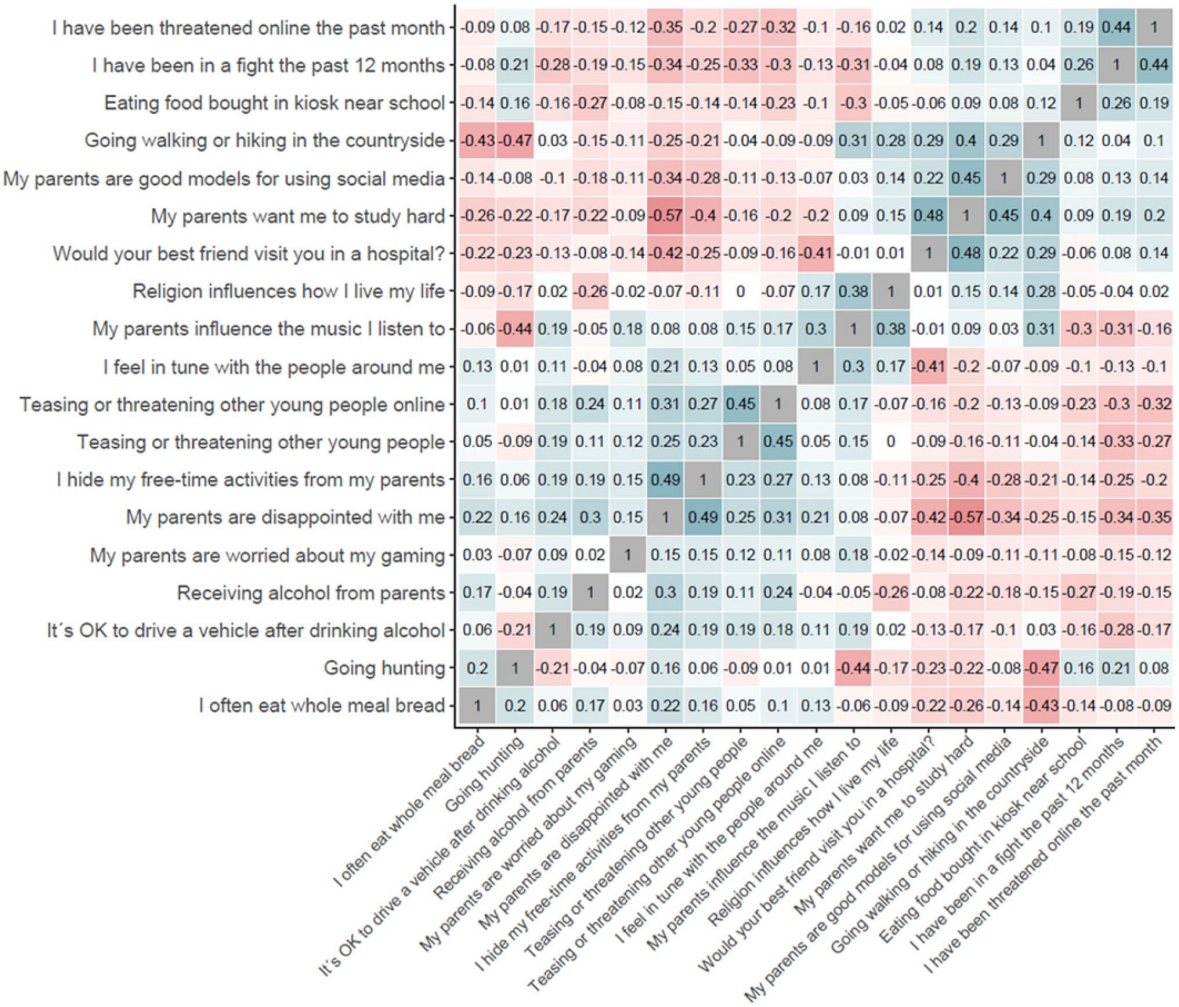
Sorted correlation matrix of most important items

In the context of this study, EFA was performed to investigate the relationships among the important variables identified by the machine learning model and to explore if they can be organized into fewer clusters representing distinct risk or protective factors. Figure 2 shows the correlation matrix of the important items. According to this figure, some of the items show adequate correlations above the absolute value of 0.4, whereas the rest of the correlations are low. For instance, items asking, *“My parents are worried about my gaming,”* “*It is OK to drive a vehicle after drinking alcohol,” “Receiving alcohol from parents,”* and *“Going hunting”* had no correlation above 0.3 with any of the other items. However, the KMO measure of sampling adequacy had a mean of 0.8 and the Bartlett’s test of sphericity was significant (χ^2^(171) = 44,829.74, *p* < 0.0001) and thus, the minimum requirements for feasibility of EFA were verified.

The parallel analysis suggested two factors, grouping items that have externalizing aspects in one factor and other items such as relationships with parents, and interpersonal relationships, physical activities, healthy diet, and religiosity in another. The 2-factor model, however, showed poor model fitness and did not meet the minimum requirements even for a partial fit (RMSEA = 0.094, TLI = 0.68, χ^2^(134) = 11,451.69, *p* < 0.0001).

Thematically, we grouped the identified important items into 4 clusters of 1) positive parenting (e.g., “*I hide my free-time activities from my parents*”, “*My parents are good models for using social media*”, “*My parents want me to study hard*”), 2) externalizing behavior (e.g., “*Teasing or threatening other young people*”, and “*I have been in a fight the past 12 months*”), 3) interpersonal relationships with parents and peers (e.g., “*My parents are disappointed with me*”, “*Would your best friend visit you in a hospital?*”), 4) lifestyle, including physical activities, hobbies, and healthy nutrition. Next, we performed an EFA by suggesting a 4-factor solution to examine whether the resulting model yielded a good model fit and reflected our subjective grouping of the important predictors. The 4-factor model showed partially good fitness (RMSEA = 0.068, TLI = 0.83, χ^2^(101) = 4613.02, *p* < 0.0001), where the RMSEA indicated a good fitness, but the TLI was below the acceptable level. As shown in Table 3, the 4-factor model continued to group the externalizing problems into a single factor. However, it distinguished items related to parental monitoring and interpersonal relationships with parents and peers from items related to physical activities and nutrition. Therefore, the 4-factor model not only provided better model fitness compared to the 2-factor model, but also, differentiated psychological, individual, interpersonal, and environmental factors from one another, indicating the importance of integrating information from various domains in studying extremism.

**Table 3:** The four-factor EFA model based on important predictors according to the model’s gain in accuracy

**Important predictors of support for political violence**	**Externalizing**	**Parental monitoring & relationships**	**Physical activities & nutrition**	**Others**
*My parents are disappointed with me*		0.63		
*I appreciate my parents’ opinions about music*				0.71
*I hide my free-time activities from my parents*		0.41		
*How often do you tease or threaten other young people?*	0.47			
*How often have you been in a fight the past 12 months?*	-0.63			
*I feel in tune with the people around me*				-0.49
*How much does religion influence your life?*				0.50
*How often do you eat food bought in kiosk near school?*	-0.46			
*How often do you eat whole meal bread?*			-0.39	
*I have been threatened online the past month*	-0.50			
*It’s OK to drive a vehicle after drinking alcohol*	0.38			
*How often do you tease or threaten other young people online?*	0.50			
*My parents are good role models for using social media*		-0.36		
*Going walking or hiking in the countryside*			0.95	
*How often do you receive alcohol from your parents?*	0.40			
*How often do you go hunting?*			-0.38	
*How many friends would visit you if you were hospitalized?*		-0.68		
*My parents want me to study hard*		-0.67		

Items that had factor loadings below 0.3 are excluded from the table

## Discussion

We explored a multitude of items from psychological, societal, environmental, and lifestyle domains to identify the most important risk and protective factors associated with adolescent extremism. The resulting machine learning classification model suggests that a comprehensive integration of mental health, well-being, family and school environment, and lifestyle factors can provide ample information to estimate the risk of extremism with a notable accuracy. Similar to finding of Nivette et al. (2022), our study found that the prevalence of extremist attitudes was significantly higher among younger adolescents, suggesting a decline in prevalence of extremist attitudes as adolescents transition into early adulthood. Next, we identified the most influential predictors within our model and explored the potential factorial structure between them. Provisionally, we clustered the identified predictors into four overarching groups encompassing 1) positive parenting, 2) externalizing behavior, 3) interpersonal relationships, and 4) lifestyle factors, including nutrition and physical activities. The EFA showed partial compatibility with our proposed factors, clustering items related to externalizing behavior, interpersonal relationships with parents and parental monitoring, and physical activities and nutrition together. The EFA also highlights the significance of the family as the key factor related to adolescents’ extremism. Family plays a pivotal role in protecting adolescents from violence, delinquency, and extremism, primarily through positive parenting practices and fostering an interest in education (Boehnke et al., 1998; Lösel & Bender, 2017). Our study provides further evidence of the significance of these elements, as they emerged as prominent predictors in our research.

The items that composed the externalizing factor in our model touched on both disinhibited and antagonism components of externalizing problems, including behaviors such as bullying, fighting, and threatening. Although items pertaining to interpersonal problems did not group well with the externalizing factor in the EFA, they emerged as important predictors in our study*.* Literature reviews show that bullying in school is among the most robust predictors of delinquency, violence, and various forms of anti-social behavior in adulthood. Moreover, the effect is even more pronounced for physical violence and direct bullying, surpassing the effect associated with verbal or indirect bullying (Bender & Lösel, 2011; Ttofi et al., 2011, 2012). Interpersonal problems with parents and peers are proposed to relate to the development of extremism, which provides the means to satisfy relational needs and the sense of belonging (Harpviken, 2020). Socialization and development of social skills in children, which enables them to initiate friendships with peers and engage in group activities, is also known to be negatively associated with delinquency and extremism (Harpviken, 2021; Lösel & Beelmann, 2003).

Another factor that emerged from the EFA was the importance of physical activities and nutrition. Adolescents’ poor diet can be a sign of disregard for their own health and is considered a key to their well-being (Laski, 2015). In addition, physical activities, along with interpersonal connections are theorized to be key components of well-being (Aked, 2011; Aked et al., 2008). Recent reviews emphasize that well-being is positively correlated with the adaptation of a healthier lifestyle, incorporating more physical activities and healthier diet (Gilbert et al., 2019; Kim et al., 2008; Rand et al., 2017). Additionally, adhering to a healthier diet is related to stronger social support (Kaiser et al., 2016; Kim et al., 2008), hence, good nutrition may signal a supportive environment conductive to adolescent well-being.

Overall, our findings underline that a narrow focus on risk factors in extremism research might neglect other significant aspects of adolescent development that could play a role in the emergence of extremism. Positive parenting approaches, well-being, externalizing behavioral issues, and lifestyle choices collectively form a comprehensive foundation to which a machine learning model can accurately assess the risk of extremist attitudes. The diversity of the important predictors in our results underline different domains and hence, support the General Strain Theory (Agnew, 2010), which conceptualizes extremism as a consequence of various psycho-socio-environmental strains.

In our study, while externalizing behavior, victimization, and substance use were identified as key predictors of extremism, other mental health problems such as anxiety, depression, low self-esteem, and eating disorders did not emerge as important predictors of adolescent extremism. After analyzing hundreds of psycho-socio-environmental variables together, this finding remains noteworthy in its own right. As depicted in Table 1, the prevalence of extremist attitudes greatly varies based on gender and age and to a certain extent, socio-economic status. However, in our study only age emerged as an important predictor, while gender and socio-economic status did not emerge as important predictors. Externalizing behavior, such as aggression and rule-breaking, tends to be more prevalent among boys, while interpersonal problems, such as difficulties in relationships, are more prevalent among girls (Bongers et al., 2004; Leadbeater et al., 1999). Similarly, socio-economic disparities can manifest in various factors that emerged as important predictors. For example, a systematic review has indicated that health behaviors, such as maintaining a healthy diet and regular exercise account for some of the socio-economic disparities in health outcomes (Petrovic et al., 2018). Therefore, much of the observed gender differences and socio-economic inequalities can be explained by other psycho-socio-environmental items, rendering basic demographic information less valuable to the model. However, age emerged as an important predictor among 550 items analyzed, indicating that the unique contribution of age information to the model could not be better explained by other items. Early adolescence, in particular, is a phase marked by experimentation and risk-taking, which could make young people more susceptible to extremist behaviors (Steinberg, 2008). In addition, during this development stage, self-control and coping skills are less developed, making adolescents more vulnerable to extremist attitudes and indoctrination (Nivette et al., 2022). Therefore, a lower prevalence of extremist attitudes among older adolescents is in line with the literature (Bakker, 2006).

However, our results contradict those of Bhui et al. (2014), who found an association between support for political violence and depression in a narrow group of immigrants in Great Britain. Furthermore, our study does not provide evidence to support the assertion made by Coid et al. (2016) regarding potential relationship between depression, neighborhood deprivation, and extremist views. Our model incorporated a comprehensive range of psycho-socio-environmental items, which allowed us to account for interactions between numerous items that might influence extremist attitudes. When considering a broader context, it's plausible that the influence of factors such as depression and neighborhood deprivation on extremist views becomes less prominent and can be better accounted for by other items. Our findings emphasize the need for comprehensive, multi-dimensional approaches in studying the risk and protective factors associated with extremist attitudes among adolescents.

## Limitations and Strengths

Although our study leveraged a nationally representative sample with hundreds of variables, the data collected relied on self-reported and retrospective accounts. Therefore, the identified important items may not necessarily serve as accurate predictors of extremism. In the same vein, from the dataset and the design of this work, no causal reference can be made regarding the relationship between the important items and the emergence of extremism. Nevertheless, the high accuracy of the model in predicting extremist attitudes along with its compatibility with the literature, suggests that the identified risk and protective factors are among the key factors contributing to adolescent extremist attitudes. In addition, the data came solely from Norwegian adolescents and thus, the identified key predictors of extremism might not be generalizable to other cultures or older adults. However, based on emerging research on extremism suggesting that individuals who endorse violence in different contexts may have some psychological factors in common (Obaidi et al., 2018a, b, c), we expect that our findings could be generalized much more broadly. A further limitation of our study is related to the nature of the lifestyle and environmental items included in our survey data. These measurements were comparatively crude, designed to capture general behaviors and habits among Norwegian adolescents. However, we deliberately included these measurements in our analysis to foster a holistic understanding of adolescents’ extremist attitudes across diverse social domains. Interestingly, these lifestyle items played a prominent role in our data analysis, suggesting that aspects such as hobbies and diets can provide unique insights into the lives of adolescents that are not captured by hundreds of psycho-socio-demographic items included in the survey.

Nevertheless, our study also had several strengths to make a unique contribution to the field. First, the study was conducted on high school students, providing information about the prevalence of extremist attitudes among ordinary adolescents rather than focusing solely on individuals with a history of violence, thus addressing a gap in the literature of extremism (Grønnerød et al., 2016). Our findings illuminate how factors from diverse domains may relate and more importantly, complement each other in elucidating extremism. This resonates with emergent empirical and theoretical insights that advocate for a more parsimonious model of extremism. Such a model would need to consider a broad range of analysis levels and incorporate a multitude of unique predictors. This includes integrating objective measures (Jacques & Taylor, 2009), social psychological dimensions (Obaidi et al., 2019; Tausch et al., 2011) and individual level variables (Obaidi et al., 2023a, b, c; Sarour & El Keshky, 2022). Lastly, our study is the first to rank the importance of different risk and protective factors related to adolescents’ extremism, while controlling for numerous of items.

## Conclusion

In summary, our findings underscore the crucial role of positive parenting in relation to adolescent extremism, as reflected in the highest-ranked items. This suggests that future research should investigate whether bolstering parenting skills could provide an effective preventative measure against the emergence of extremist attitudes in adolescents. Previous studies have highlighted that positive parenting practices, characterized by warmth, support, and monitoring, are linked to a decreased likelihood of adolescents engaging in risky behaviors, such as criminal activity and unsafe sexual behaviors (Hoeve et al., 2009; Sidze & Defo, 2013). Items pertaining to problems in interpersonal relationships with parents and peers emerged as the second important group of predictors, followed by externalizing problems as the third important predictor of adolescents’ extremism. Aspects of adolescents’ physical health and well-being were the fourth important group of predictors.

These findings supported the General Strain Theory (Agnew, 2010), positing that various risk and protective factors from different domains play a role in preventing or promoting extremism. Our study also demonstrated that by conducting a holistic item analysis and without imposing linear relationships or a particular pre-specified model or variable selection, various psychological factors provide rich information, leading to accurate prediction and classification of adolescents’ extremism. Therefore, it is important to consider not only mental disorders, but also positive and negative aspects of adolescents’ mental health, which can provide significant information about adolescents’ extremist attitudes. Interventions that focus on single factors in isolation are less likely to have a preventive impact compared to a more holistic approach to prevention, addressing a combination of factors (Bjørgo, 2016a, b). Some of the predictors for extremist attitudes analyzed in this study are more amenable to intervention and positive change than others. Particularly, our results support the recent extremism prevention programs that prioritize adolescents’ well-being (Benjamin et al., 2021; Koirikivi et al., 2021). Building upon our study, further analysis can use our findings as a foundation to identify holistic strategies for preventive intervention.

## Data Availability

The data used in this study is not publically available. However, researchers can apply for access to the data via https://www.ungdata.no/.

## Footnotes

1 Visit www.ungdata.no
